# Is fast-food consumption a problem among adolescents in Malaysia? An analysis of the National School-Based Nutrition Survey, 2012

**DOI:** 10.1186/s41043-021-00254-x

**Published:** 2021-07-16

**Authors:** Cheong Siew Man, Lim Kuang Hock, Chan Ying Ying, Kee Chee Cheong, Lim Kuang Kuay, Teh Chien Huey, Azli Baharudin, Nur Shahida Abdul Aziz

**Affiliations:** 1 Centre for Nutrition Epidemiology Research, Institute for Public Health, National Institutes of Health, Ministry of Health Malaysia, No.1, Jalan Setia Murni U13/53, Section U13, Setia Alam, 40170 Shah Alam, Selangor Malaysia; 2 Special Research Centre, Institute for Medical Research, National Institutes of Health, Ministry of Health Malaysia, Kuala Lumpur, Malaysia; 3 Centre for Family Health Research, Institute for Public Health, National Institutes of Health, Ministry of Health Malaysia, Shah Alam, Selangor Malaysia; 4 Sector for Biostatistics and Data Repository, National Institutes of Health, Ministry of Health Malaysia, Shah Alam, Selangor Malaysia; 5 Centre for Occupation Health Research, Institute for Public Health, National Institutes of Health, Ministry of Health Malaysia, Shah Alam, Selangor Malaysia; 6 Biomedical Research Management, Strategy and Innovation Unit, Institute for Medical Research, National Institutes of Health, Ministry of Health Malaysia, Shah Alam, Selangor Malaysia

**Keywords:** Fast-food intake, Schooling adolescents, Healthy food choices, Dietary habit

## Abstract

**Background:**

Fast-food consumption is an unhealthy dietary behaviour because it increases the risk of diet-related chronic diseases. We aimed to investigate factors associated with fast-food consumption, namely sociodemographic characteristics, body mass index-for-age, meal away from home habit, and intake of various food groups among adolescents in Malaysia.

**Methods:**

We analysed data from the National School-Based Nutrition Survey (NSNS). The NSNS was a nationwide, cross-sectional survey. Multiple-stage stratified cluster random sampling method was applied to obtain a representative sample of adolescents’ population. This study recruited adolescents aged 10–18 years who were attending schools. Pre-tested self-administered questionnaires in Malay language were used to obtain relevant information. Frequency of fast-food consumption per week was classified into three groups: “consumed fast-food four to seven days”, “consumed fast-food one to three days”, and “did not consume fast-food”. Intake of food groups was assessed by self-administered food frequency questionnaire. Descriptive and complex sample multinomial logistic regression analyses were performed in data analysis.

**Results:**

A total of 26,383 from 40,012 selected adolescents completed all the relevant questions for this study. Of those surveyed, 13.5% of the respondents consumed fast-food 4 to 7 days, 69.3% of the respondents consumed fast food 1 to 3 days, and 17.2% of them did not consume fast-food in a typical week. Frequency of fast-food consumptions (4 to 7 days and 1 to 3 days per week) was significantly associated with age; sex; ethnicity; locality of schools; frequency of eating out; and not consuming recommended intake of cereals or grains, vegetables, and meat or poultry or eggs.

**Conclusion:**

In conclusion, age; sex; ethnicity; locality of schools; frequency of eating out per week; imbalanced intake of cereals or grains, meat, or poultry or eggs; and inadequate vegetable intake were significantly associated with fast-food consumption among adolescents in Malaysia. The findings of this study will be useful for policy makers in promoting healthy food choices among adolescents in Malaysia.

## Introduction

Fast food is defined as “hot food such as hamburgers that is quick to cook or is already cooked and is therefore served very quickly in a restaurant” [1]. The fast-food industry has spread the fast-food culture worldwide: fast-food outlets and vendors have managed to expand in both developed and developing countries despite the increasing awareness of the adverse health effects associated with a diet high in fat, salt, and sugar. Over the past three decades, rapid economic development, urbanisation, and the influence of Western cultures have drastically changed the lifestyles of Malaysians. Malaysia first saw marked changes in dietary habits because of its rapid economic growth and changes in occupational patterns in the 1980s [2], which led the fast-food industry to experience heavy growth in the 1990s [3]. In the 2000s, the Department of Statistics Malaysia found that the number of fast food restaurants in Malaysia increased from 1621 in 2010 to 2597 in 2015 [4].

Adolescents who consumed unhealthy diet such as higher energy-dense food and inadequate intake of fruits and milk were more likely to consume fast-food frequently [5]. Poor dietary habits among adolescents can lead to excessive weight gain and increase the risk of obesity in adulthood because dietary habits that are established during adolescence could persist to adulthood [6, 7]. Relevant literature from other countries revealed that fast-food consumption is prevalent in younger people, higher household income groups, adults with higher body mass index (BMI), and men [8–10]. In China, prevalence of fast-food consumption among Chinese adolescents aged 13–17 years increased rapidly from 17.9 to 26.3% between 2004 and 2009 [11]. Therefore, it would be interesting to investigate fast-food consumption pattern among adolescents in Malaysia.

Despite growing evidence of the increasing trend in fast-food consumption among adolescents, majority of the fast-food consumption studies in Malaysia were mainly conducted among children and adults [12, 13]. Moreover, local and national studies that examine the factors associated with fast-food consumption among adolescents are scarce. To the best of our knowledge, there was only one study that described fast-food intake habit among children aged 2 to 12 years old with responses from caregivers or parents. The study reported that 9.7% children in Malaysia ate fast-food at least once a week [13]. Therefore, the present study aims to investigate the factors associated with fast-food consumption in terms of sociodemographic characteristics, locality of schools, BMI, frequency of eating out, and intake of various food groups among adolescents in Malaysia. The findings of this study are essential for providing supporting data to policy makers and relevant government agencies in promoting healthy eating habit among adolescents.

## Methodology

### Data sources

Data from the National School-Based Nutrition Survey (NSNS) was used for the present analysis. The NSNS was a sub survey of the 2012 National School-Based Health Survey (NSHS) in Malaysia [14]. This nationwide survey was conducted from February to April 2012. Multiple-stage stratified cluster random sampling was used to obtain a representative sample of adolescents aged 10–18 years from all states in Peninsular and East Malaysia. The sampling frame consisted of all primary and secondary schools under the Ministry of Education Malaysia. The first stage of sampling was a selection of schools with probability proportional to school enrolment size nationwide. In total, 234 schools were selected to participate in this study. The second stage of sampling was random selection of classrooms from each selected school. The third stage of sampling was random selection of students from each selected class to provide information on intake of various food groups by answering a food frequency questionnaire (FFQ). All students in the selected classrooms were given informed consent forms prior to recruitment into this study. Parents or caregivers of primary school students were given consent forms, and secondary school students were given self-administered assent forms. A total of 40,011 selected adolescents participated in the NSNS with a student response rate of 90.5%. Only 26,588 respondents were randomly selected to answer the FFQ in order to provide information on intake of various food groups. As a result of the selection, 26,383 students completed FFQ with the response rate of 99.2%.

### Variable definitions

Frequency of fast-food consumption was assessed using the question, “Normally, how many days in a week do you eat fast food which was bought from fast-food restaurants, such as burgers, pizza, hotdogs, nuggets, fried chicken, French fries, and other similar things?” The answers provided by the respondents ranged from 0 to 7 days. The dependent variable was classified into three categories which were adolescents who consumed fast food 4 to 7 days, adolescents who consumed fast food 1 to 3 days, and adolescents who did not consume fast food in a week.

The independent variables were age, sex, locality of schools, ethnicity, weight status, frequency of eating out per week, and intake of food groups per day. Age was grouped as 10–12 years, 13–15 years, and 16–18 years. Locality of schools was determined according to the geographical areas in both Peninsular Malaysia (Northern Zone, Central Zone, Southern Zone, and East Coast) and East Malaysia. The Northern Zone included the states of Perlis, Kedah, Pulau Pinang, and Perak. States in the Central Zone were Selangor, Putrajaya Federal Territory, and Kuala Lumpur Federal Territory. The Southern Zone consisted of Malacca, Negeri Sembilan, and Johor. The East Coast comprised of the states of Pahang, Terengganu, and Kelantan. Finally, East Malaysia was the states of Sabah and Sarawak. Ethnicities in Malaysia were grouped into Malay, Chinese, Indian, Indigenous from East Malaysia, and other ethnicities.

Body weight and height of respondents were measured twice using TANITA HD-319 electronic weighing scales (Tanita Corp., Tokyo, Japan) and SECA 213 stadiometer (SECA GmbH & Co. KG, Hamburg, Germany) to ± 0.1kg and ± 0.1cm, respectively. The average values of body weight and body height were used for BMI-for-age calculation. BMI-for-age z-score was calculated using the AnthroPlus software. The weight status of students was classified into four categories: underweight (<−2SD z-scores), normal (≥−2SD to <+1SD z-scores), overweight (≥+1SD to <+2SD z-scores), and obese (≥+2SD z-scores) based on WHO’s growth reference for children aged 4 to 19 [15]. Frequency of eating out per week was coded as “more than six times”, “three to five times”, “one to two times”, and “never” based on the multiple choice question: “In a week, how often do you eat out?”

A pretested, self-administered semi-quantitative FFQ for adolescents containing 135 items was developed by panel of experts to obtain information on food consumption based on seven food groups (i.e. cereals or grains, fruits, vegetables, milk or dairy products, meat or poultry or eggs, fish, and legumes). The intake of various food groups per day obtained from the FFQ was translated into the Malaysian Dietary Guideline (MDG) standard serving size [16]. According to the MDG, the recommended intake of cereals or grains was eight to 11 servings per day. For meat or poultry or eggs, the recommended intake was 0.5 to two servings per day, and it is recommended to consume at least three servings of vegetables per day.

### Data analysis

Data analyses were conducted using SPSS version 22 (SPSS IBM, New York, USA). Weighing was applied to take into account the complex study design and non-response rate. Descriptive statistics was used to describe the sociodemographic characteristics and frequency of fast-food consumption among adolescents. Factors associated with frequency of fast-food consumption were analysed using complex sample multinomial logistic regression analysis. All statistical analyses were carried out at 95% confidence interval or *p-*value < 0.05.

## Results

As shown in Table 1, there were almost equal number of girls and boys in the sample, and majority of the respondents (60.2%) were Malay adolescents. Slightly more than half of the respondents (58.0%) were of 13–15 years age group, and more than one quarter of them (28.0%) were overweight (14.9%) or obese (13.1%). Overall, 13.5% of the respondents consumed fast-food 4 to 7 days, 69.3% of the respondents consumed fast-food 1 to 3 days, and 17.2% of them did not consume fast-food in a typical week. Table 2 shows that the prevalence of adolescents who consumed fast-food 4 to 7 days were significantly higher among Malay adolescents (15.1%) compared with Indian adolescents (10.7%) and Chinese adolescents (7.3%). The prevalence of adolescents who consumed fast-food 4 to 7 days were significantly lower among adolescents living in Northern zone (10.2%) compared with adolescents living in other zones (ranged from 13.1 to 17.0%), and those who never eat out (9.9%) compared with those who eat out at least three times per week (ranged from 20.1 to 26.2%). Table 2 also shows that the prevalence of consuming fast food 1 to 3 days was significantly higher among girls (71.3%) compared with boys (67.3%), Malays adolescents (70.9%) compared with Chinese adolescents (66.2%), those eating out one to three times per week (73.0%) compared with those eating out more than three times per week (ranged from 59.0 to 66.7%).

**Table 1:** Characteristics of study respondents

Characteristics	Unweighted count (n)	% (95% CI)
**Age group**		
16–18 years	5724	22.9% (21.1–24.7)
13–15 years	15,615	58.0% (56.1–59.8)
10–12 years	5044	19.2% (17.5–20.9)
**Sex**		
Girls	13,078	49.8% (47.8–51.9)
Boys	13,305	50.2% (48.1–52.2)
**Ethnicity**		
Malay	17,618	60.2% (55.4–64.9)
Indian	1553	6.9% (5.6–8.6)
Indigenous from East Malaysia	2123	10.6% (8.7–13.0)
Other	452	1.8% (1.3–2.7)
Chinese	4625	20.3% (16.3–25.1)
**BMI-for-age**		
Underweight	1984	7.6% (7.1–8.1)
Overweight	3962	14.9% (14.3–15.6)
Obese	3433	13.1% (12.5–13.8)
Normal	16,924	64.5% (63.5–65.5)
**Locality of schools**		
Central zone	3453	21.9% (18.4–25.7)
Southern zone	5319	19.5% (16.8–22.4)
East coast zone	4908	17.2% (15.1–19.5)
East Malaysia	3494	17.9% (15.2–20.8)
Northern zone	7230	23.7% (20.9–26.7)
**Frequency of fast food consumption per week**		
4–7 days	3637	13.5% (12.8–14.2)
1–3 days	18,232	69.3% (68.4–70.1)
0 day	4469	17.2% (16.3–18.2)
**Eating out per week**		
> 6 times	1299	5.1% (4.6–5.6)
3–5 times	3814	14.0% (13.1–15.0)
1–2 times	16,708	63.2% (62.2–64.3)
Never	4465	17.7% (16.5–19.0)

**Table 2:** Prevalence of fast-food consumption among adolescents by sociodemographic characteristics, BMI, and eating out behaviour

Variables	Frequency of fast food consumption per week					
	4–7 days (*n* = 3637)		1–3 days (*n* = 18,232)		0 day (*n* = 4467)	
	%	95% CI	%	95% CI	%	95% CI
**Age group**						
16–18 years	12.8	(11.5–14.2)	69.4	(67.8–71.0)	17.8	(16.3–19.4)
13–15 years	13.1	(12.3–14.0)	70.0	(69.0–71.1)	16.8	(15.7–18.0)
10–12 years	15.4	(14.0–17.0)	66.7	(64.8–68.6)	17.8	(16.3–19.5)
**Sex**						
Girls	13.1	(12.2–14.0)	71.3	(70.2–72.4)	15.6	(14.5–16.8)
Boys	13.9	(13.0–14.9)	67.3	(66.1–68.4)	18.8	(17.7–20.0)
**Ethnicity**						
Malay	15.1	(14.2–16.0)	70.9	(69.9–71.9)	14.0	(13.2–15.0)
Indian	10.7	(9.1–12.6)	69.8	(67.2–72.4)	19.5	(17.0–22.2)
Indigenous from East Malaysia	17.9	(15.6–20.4)	65.9	(63.2–68.6)	16.2	(13.9–18.7)
Other	14.3	(10.5–19.2)	68.2	(64.0–72.2)	17.5	(13.5–22.3)
Chinese	7.3	(6.4–8.3)	66.2	(64.2–68.1)	26.5	(24.6–28.5)
**Locality of schools**						
Central zone	13.1	(11.5–15.0)	70.8	(68.9–72.7)	16.1	(14.1–18.2)
Southern zone	13.1	(11.5–14.9)	68.7	(67.0–70.4)	18.2	(16.3–20.4)
East coast zone	15.3	(13.9–16.8)	68.4	(66.6–70.2)	16.3	(14.5–18.2)
East Malaysia	17.0	(15.2–19.0)	67.0	(64.8–69.1)	16.0	(14.0–18.2)
Northern zone	10.2	(9.0–11.4)	70.6	(68.8–72.3)	19.2	(17.2–21.5)
**BMI-for-age**						
Underweight	12.7	(11.0–14.7)	70.7	(68.2–73.0)	16.6	(14.6–18.9)
Overweight	14.0	(12.8–15.4)	68.8	(67.1–70.5)	17.1	(15.7–18.7)
Obese	13.9	(12.3–15.6)	68.9	(66.8–70.9)	17.3	(15.5–19.1)
Normal	13.4	(12.6–14.2)	69.3	(68.4–70.2)	17.3	(16.3–18.4)
**Eating out per week**						
>6 times	26.2	(23.1–29.5)	59.0	(55.6–62.3)	14.8	(12.6–17.4)
3–5 times	20.1	(18.3–22.0)	66.7	(64.9–68.5)	13.2	(11.7–14.9)
1–2 times	12.0	(11.1–12.8)	73.0	(72.1–73.9)	15.0	(14.0–16.1)
Never	9.9	(8.7–11.2)	61.4	(59.3–63.4)	28.7	(26.7–30.8)

Table 3 shows that adolescents aged 16–18 years old were less likely to consume fast-food 1 to 3 days per week (aOR 0.46, 95% CI 0.26, 0.82) compared with younger age group of 10–12 years. Girls were more likely to consume fast-food (aOR 1.44, 95% CI 1.26–1.66 for 4 to 7 days per week and aOR 1.44, 95% CI 1.30–1.61 for 1 to 3 days per week) compared with boys. Non-Chinese adolescents (ranged from aOR 2.23 to 4.09) were more likely to consume fast-food 4 to 7 days per week compared with Chinese adolescents. At the same time, Malay adolescents (aOR 1.99, 95% CI 1.76–2.26), Indigenous from East Malaysia adolescents (aOR 1.27, 95% CI 1.04–1.57), and adolescents with other ethnicities (aOR 1.69, 95% CI 1.20–2.38) were more likely to consume fast-food 1 to 3 days per week compared with Chinese adolescents.

**Table 3:** Factors associated with fast-food consumption among adolescents

Independent variables	Fast-food consumption											
	4–7 days						1–3 days					
	Crude odds ratio	(95% CI)	p	Adjusted odds ratio	(95% CI)	p	Crude odds ratio	(95% CI)	p	Adjusted odds ratio	(95% CI)	p
**Age group**												
16–18 years	0.83	(0.68–1.01)	0.056	0.80	(0.63–1.02)	0.073	1.04	(0.91–1.20)	0.558	0.46	(0.26–0.82)	**0.008**
13–15 years	0.90	(0.77–1.06)	0.216	0.87	(0.70–1.07)	0.180	1.11	(0.98–1.26)	0.093	1.08	(0.92–1.28)	0.356
10–12 years	1.00			1.00			1.00		1.000	1.00		
**Sex**												
Girls	1.14	(1.01–1.28)	0.036	1.44	(1.26–1.66)	**<0.001**	1.28	(1.16–1.41)	<0.001	1.45	(1.30–1.61)	**<0.001**
Boys	1.00			1.00			1.00			1.00		
**Ethnicity**												
Malay	3.88	(3.22–4.66)	<0.001	4.09	(3.31–5.07)	**<0.001**	2.02	(1.79–2.28)	<0.001	1.99	(1.76–2.26)	**<0.001**
Indian	1.98	(1.50–2.62)	<0.001	2.23	(1.63–3.03)	**<0.001**	1.43	(1.17–1.75)	<0.001	1.27	(1.04–1.57)	**0.023**
Indigenous from East Malaysia	4.00	(3.02–5.30)	<0.001	2.59	(1.82–3.70)	**<0.001**	1.63	(1.34–1.99)	<0.001	1.32	(0.96–1.81)	0.085
Other	2.96	(1.79–4.87)	<0.001	3.69	(2.42–5.62)	**<0.001**	1.56	(1.16–2.11)	0.004	1.69	(1.20-2.38)	**0.003**
Chinese	1.00			1.00						1.00		
**Locality of schools**												
Central zone	1.55	(1.14–2.10)	0.005	.37	(1.07–1.76)	**0.014**	1.20	(0.99–1.47)	0.069	1.16	(1.01–1.33)	**0.040**
Southern zone	1.36	(1.01–1.84)	0.043	1.29	(1.00–1.65)	0.050	1.03	(0.85–1.24)	0.772	1.01	(0.88–1.17)	0.849
East coast zone	1.79	(1.37–2.34)	<0.001	1.25	(0.96–1.63)	0.092	1.15	(0.95–1.38)	0.149	0.97	(0.82–1.15)	0.734
East Malaysia	2.02	(1.51–2.70)	<0.001	1.95	(1.45–2.62)	**<0.001**	1.14	(0.93–1.40)	0.196	1.25	(0.98–1.60)	0.073
Northern zone	1.00			1.00			1.00			1.00	1.00	
**BMI-for-age**												
Underweight	0.99	(0.80–1.23)	0.941	-	-		1.06	(0.91–1.24)	0.434	-	-	
Overweight	1.06	(0.92–1.24)	0.420				1.01	(0.90–1.13)	0.922			
Obese	1.04	(0.89–1.23)	0.618				1.00	(0.89–1.13)	0.988			
Normal	1.00						1.00					
**Eating out per week**												
>6 times	5.13	(3.92–6.72)	<0.001	9.16	(6.67–12.58)	**<0.001**	1.86	(1.49–2.33)	<0.001	2.58	(2.00–3.31)	**<0.001**
3–5 times	4.42	(3.50–5.51)	<0.001	6.39	(5.07–8.06)	**<0.001**	2.37	(2.01–2.78)	<0.001	2.92	(2.44–3.51)	**<0.001**
1–2 times	2.31	(1.94–2.76)	<0.001	2.57	(2.11–3.12)	**<0.001**	2.27	(2.04–2.54)	<0.001	2.33	(2.06–2.63)	**<0.001**
Never	1.00			1.00			1.00			1.00		
**Cereals/grains**												
Inadequate	0.46	(0.36–0.58)	<0.001	0.67	(0.52–0.88)	**0.003**	0.60	(0.53–0.63)	<0.001	0.73	(0.64–0.83)	0.727
Exceed	1.91	(1.68–2.18)	<0.001	1.34	(1.17–1.54)	**<0.001**	1.34	(1.21–1.49)	<0.001	1.12	(0.98–1.27)	1.115
Normal	1.00			1.00			1.00			1.00		
**Fruits**												
Inadequate	0.64	(0.56–0.72)	<0.001	1.02	(0.88–1.19)	0.784	0.74	(0.68–0.81)	<0.001	0.96	(0.87–1.05)	0.348
Normal	1.00			1.00			1.00			1.00		
**Vegetables**												
Inadequate	1.12	(0.88–1.41)	0.366	1.60	(1.07–2.39)	**0.022**	1.07	(0.90–1.27)	0.434	1.35	(1.06–1.73)	**0.016**
Normal	1.00			1.00			1.00			1.00		
**Milk/dairy products**												
Inadequate	0.66	(0.56–0.79)	<0.001	0.98	(0.78–1.23)	0.862	0.81	(0.71–0.93)	0.003	1.07	(0.92–1.25)	0.366
Exceed	1.31	(0.91–1.90)	0.148	0.89	(0.49–1.61)	0.692	1.14	(0.83–1.55)	0.422	1.23	(0.78–1.94)	0.373
Normal	1.00			1.00			1.00			1.00		
**Meat/poultry/eggs**												
Inadequate	0.45	(0.35–0.59)	<0.001	0.53	(0.38–0.73)	**<0.001**	0.54	(0.46–0.62)	<0.001	0.66	(0.55–0.78)	**<0.001**
Exceed	2.58	(2.28–2.91)	<0.001	1.99	(1.70–2.32)	**<0.001**	1.48	(1.35–1.61)	<0.001	1.26	(1.13–1.40)	**<0.001**
Normal	1.00			1.00			1.00			1.00		
**Fish**												
Inadequate	1.38	(0.75–2.56)	0.305	1.28	(0.62–2.63)	0.507	1.19	(0.78–1.84)	0.419	1.38	(0.85–2.24)	0.195
Exceed	2.39	(1.29–4.40)	0.005	1.26	(0.61–2.62)	0.532	1.65	(1.08–2.54)	0.022	1.40	(0.86–2.29)	0.178
Normal	1.00			1.00			1.00			1.00		
**Legumes**												
Inadequate	0.78	(0.67–0.90)	0.001	1.03	(0.85–1.25)	0.778	0.78	(0.70–0.88)	<0.001	0.95	(0.83–1.10)	0.495
Exceed	1.27	(1.07–1.51)	0.007	0.85	(0.68–1.07)	0.165	1.08	(0.94–1.23)	0.283	0.91	(0.77–1.08)	0.280
Normal	1.00			1.00			1.00			1.00		

Dependent variable: did not consume fast food per week as reference category

Adolescents from central zone (aOR1.37, 95% CI 1.07–1.76) and East Malaysia (aOR 1.95, 95% CI 1.45–2.62) were more likely to consume fast food 4 to 7 days per week compared with adolescents from Northern zone. Adolescents who were eating out one to two times, three to five times, and more than six times per week were more likely to consume fast food (aOR 2.57 to 9.16 for 4 to 7 days per week and aOR 2.33 to 2.92 for 1 to 3 days per week) compared with adolescents who did not eat out.

Adolescents who consumed above recommended intake of cereals or grains were more likely to consume fast food 4 to 7 days per week (aOR 1.34, 95% CI 1.17–1.54) compared with those who achieved the recommendation. Adolescents who consumed less than recommended intake of vegetables (aOR 1.60, 95% CI 1.07, 2.39) were more likely to consume fast food 4 to 7 days per week compared with their counterparts with adequate vegetable intake. Adolescents who consumed above recommended intake of meat or poultry or eggs were more likely to consume fast food (aOR 1.99, 95% CI 1.70–2.32 for 4 to 7 days per week and aOR 1.26, 95% CI 1.13–1.40) compared with adolescents who achieved the recommended intake of this food group.

## Discussion

This study revealed that older age adolescents (16–18 years old) were less likely to consume fast food compared with the younger age adolescents (10–12 years old). This may be due to older age adolescents experienced physical changes in puberty. The physical changes caused them encountered body shape problems [17]. A previous study revealed that adolescents with body image dissatisfaction were less likely to follow Western dietary pattern but choose a restrictive pattern [18]. Therefore, restriction of fast-food consumption could be part of the diet control behaviour among older adolescents with body image dissatisfaction. This study reported girls were significantly more likely to consume fast food compared with boys. The finding of this study contradicts with other studies [19, 20]. A previous study found that adolescent girls were more likely to visit fast-food outlets in special days to meet with friends or family than boys especially in city centres [21]. Another previous study disclosed that the main reasons of adolescent girls consuming fast food were enjoying the taste of fast food and favouring the convenience of the fast-food restaurants [22]. However, there is no evidence found to explain the preference of girls to consume fast food compared with boys. This study suggests future research to investigate about the gender preferences and perception of fast-food consumption which are needed in order to fill this research gap.

In this study, ethnicity was found to be significantly associated with fast-food consumption. Non-Chinese adolescents were more likely to consume fast food than Chinese adolescents. The findings from this study are also in line with a previous Malaysian study that found Chinese adolescents consumed healthier food than Malay adolescents [21]. Two possible reasons for the ethnic differences in fast-food consumption were identified. Firstly, Chinese adolescents are less likely to be influenced by unhealthy food advertising on television compared with other ethnicities since their childhood [23]. Secondly, previous study revealed that traditional Chinese parenting styles are more concerned with healthy eating practices among their children [24]. For instance, most Chinese mothers would restrict certain unhealthy foods and encourage balanced food intake for health reasons [24].

Adolescents from the Central Zone and East Malaysia were significantly more likely to consume fast food compared with those from the Northern Zone. Availability of fast-food restaurants nearby school areas and neighbourhoods were crucial factors contributing to fast-food consumption habits among adolescents [25]. In Malaysia, fast-food restaurants are always available in areas with higher density of population. Therefore, adolescents who live in Northern Zone with lower density of population [26] may have less frequent access to fast-food than adolescents from the Central Zone. Interestingly, fast-food consumption was also high in East Malaysia although most of the residential areas there were of lower density of population. A possible reason may be that there is rapid expansion of franchise fast-food chain and home-grown fast-food chain in East Malaysia after implementing the Franchise Development Program in 1992 [27]. This programme may change the dietary habits of adolescents and younger adults in East Malaysia. Future study is suggested to investigate fast-food consumption pattern in East Malaysia to better understand the fast-food intake behaviour among adolescents in East Malaysia.

The findings indicate that fast-food consumption among adolescents was not associated with BMI for age. This is in line with previous studies [11, 28, 29], although there were studies that demonstrated otherwise among adults [9, 10]. This could be due to not only fast-food consumption, but also their overall dietary intake being the main contributor to being overweight or obese among adolescents [29]. For example, products such as sugary beverages and chips from retail food stores contain similar levels of fat and sugar as in fast food [29]. Another possible explanation may be that overweight or obese adolescents tend to under-report the frequency of fast-food intake [30] due to the possibility of social desirability bias, as most people know that fast food is unhealthy food. However, other related factors that could confound the relationship between fast-food consumption and BMI, such as physical activity and body image perception, were not investigated in this study [17, 31].

In this study, fast-food consumption was associated with frequency of eating out per week. Eating out of home is increasingly common among Malaysian households since 1990s [32, 33] along with increasing household income and lifestyle changes. A Malaysian study found that 91.2% of adolescents aged between 13 and 14 years old have family meal away from home at least once a week in an urban area in Selangor [33]. Households with higher income and in urban areas have higher probability of dining away from home for all ethnicities in Malaysia [34]. The local study also reported that, among adolescents with having meal away from home habit, 41.6% of them visited fast-food outlets when they were eating outside with their families [33]. Our study suggests that eating out increases the probability of eating fast food regardless of eating with friends or family members among adolescents because fast-food outlets are considered one of the common choices for eating out. Therefore, knowledge, skill, and motivation to opt for healthy food are needed within the households in order to promote healthy eating away from home among adolescents.

This study also revealed that consumption of cereals or grains, vegetables, and meat or poultry or eggs were factors significantly associated with fast-food consumption. Adolescents who consumed excessive cereals or grains (more than 11 servings per day) and meat or poultry or eggs (more than two servings per day) were significantly more likely to consume fast food. In addition, those who consumed inadequate vegetables (less than three servings per day) were also more likely to consume fast food [16]. This finding is reasonable because chicken-based, bread-based, and fish-based fast food were the favourite fast-food items among young Malaysians [35]. This study suggests that adolescents who consumed fast food often linked to imbalanced dietary intake which may increase the risk of having non-communicable diseases among adolescents [21].

The major strength of this study is the use of a nationally representative sample to demonstrate findings applicable to the Malaysian adolescent population. In addition, the findings of this study provide new insights to the existing literature on dietary behaviour among adolescents in Malaysia. Fast-food outlets are expanding to rural areas in the recent years. This study also recruited adolescents from remote zones such as East Malaysia, adding new information on dietary behaviours of adolescents in the remote areas. There are a few limitations in this study. First, this is a cross-sectional study; therefore, it is not possible to establish cause and effect relationship in this study. Second, owing to self-recall of fast-food consumption, the data may contain self-recall bias. It is possible that frequency of fast-food consumption being over- or under-reported in this study.

## Conclusion

In conclusion, significant factors associated with fast-food consumption were age, sex, ethnicity, locality of schools, frequency of eating out, imbalanced intake of cereals or grains, meat or poultry or eggs, and inadequate vegetables intake. This study provides evidenced-based findings to help policy makers and professionals in designing more effective strategies and programmes to promote healthy food choices among adolescents in Malaysia. Despite the Ministry of Health Malaysia initiated a healthy eating strategy in fast-food restaurants to provide healthier food options to the public [36], all relevant committees should strengthen the existing strategy through reformulating fast-food products to reduce fat, salt, and sugar content as fast food is commonly consumed among adolescents.

## Data Availability

The dataset that supports the findings of this study belongs to the Ministry of Health Malaysia.

## Abbreviations

BMI: Body mass index
FFQ: Food frequency questionnaire
MDG: Malaysian Dietary Guidelines
NSNS: National School-Based Nutrition Survey
WHO: World Health Organization
